# Autoimmune polyendocrine syndrome type 1: an Italian survey on 158 patients

**DOI:** 10.1007/s40618-021-01585-6

**Published:** 2021-05-18

**Authors:** S. Garelli, M. Dalla Costa, C. Sabbadin, S. Barollo, B. Rubin, R. Scarpa, S. Masiero, A. Fierabracci, C. Bizzarri, A. Crinò, M. Cappa, M. Valenzise, A. Meloni, A. M. De Bellis, C. Giordano, F. Presotto, R. Perniola, D. Capalbo, M. C. Salerno, A. Stigliano, G. Radetti, V. Camozzi, N. A. Greggio, F. Bogazzi, I. Chiodini, U. Pagotto, S. K. Black, S. Chen, B. Rees Smith, J. Furmaniak, G. Weber, F. Pigliaru, L. De Sanctis, C. Scaroni, C. Betterle

**Affiliations:** 1 Endocrine Unit, Department of Medicine (DIMED), University of Padua, Via Ospedale Civile 105, 35128 Padua, Italy; 2 Unit of Internal Medicine, Ospedale dell’Angelo, Mestre-Venice, Italy; 3 Unit of Internal Medicine, Ospedale di Feltre, Belluno, Italy; 4 Infectivology and Clinical Trials Research Department, Bambino Gesù Children’s Hospital, IRCCS, Rome, Italy; 5 Endocrine Unit, Bambino Gesù Children’s Hospital, IRCCS, Rome, Italy; 6 Unit of Pediatrics, Department of Adulthood and Childhood Human Pathology, University of Messina, Messina, Italy; 7 Ospedale Microcitemico and Dipartimento di Scienze Biomediche e Biotecnologiche, University of Cagliari, Cagliari, Italy; 8 Unit of Endocrinology and Metabolic Diseases, Department of Advanced Medical and Surgical Sciences, University of Campania “Luigi Vanvitelli”, Naples, Italy; 9 Endocrine Unit, Department of Biomedical Internal and Specialist Medicine (DIBIMIS), Palermo University, Palermo, Italy; 10 Department of Pediatrics, Regional Hospital Vito Fazzi, Lecce, Italy; 11 Department of Mother and Child, University Federico II, Naples, Italy; 12 Pediatric Section, Department of Translational Medical Sciences, University Federico II, Naples, Italy; 13 Endocrinology, Department of Clinical and Molecular Medicine, Sant’Andrea Hospital, Sapienza University of Rome, Rome, Italy; 14 Marienklinik, General Hospital, Bolzano, Italy; 15 EU-Endo-ERN Advisory Board Member, National Coordinator Endo-ERN Pediatric (SIEDP), Padua, Italy; 16 Department of Clinical and Experimental Medicine, University of Pisa, Pisa, Italy; 17 Unit of Bone Metabolism Diseases and Diabetes, Istituto Auxologico Italiano, Department of Medical Biotechnology and Translational Medicine, University of Milan, Milan, Italy; 18 Unit of Endocrinology and Prevention and Care of Diabetes, Department of Medical and Surgical Sciences (DIMEC), University of Bologna, Bologna, Italy; 19 FIRS Laboratories RSR Ltd, Cardiff, UK; 20 Unit of Pediatrics, Vita-Salute San Raffaele University, IRCSS San Raffaele Scientific Institute, Milan, Italy; 21 Endocrine Unit, Azienda Ospedaliera-Universitaria of Cagliari, Cagliari, Italy; 22 Pediatric Endocrinology, Department of Public Health and Pediatric Sciences, Regina Margherita Children’s Hospital, University of Turin, Turin, Italy

**Keywords:** Autoimmune-poly-endocrine-candidiasis-ectodermal-dystrophy (APECED), Autoimmune Polyglandular Syndrome type 1 (APS-1), AIRE gene mutations, Chronic mucocutaneous candidiasis, Chronic hypoparathyroidism, Addison’s disease, Interferon autoantibodies

## Abstract

**Background:**

Autoimmune Polyglandular Syndrome type 1 (APS-1) is a rare recessive inherited disease, caused by AutoImmune Regulator (*AIRE*) gene mutations and characterized by three major manifestations: chronic mucocutaneous candidiasis (CMC), chronic hypoparathyroidism (CH) and Addison’s disease (AD).

**Methods:**

Autoimmune conditions and associated autoantibodies (Abs) were analyzed in 158 Italian patients (103 females and 55 males; F/M 1.9/1) at the onset and during a follow-up of 23.7 ± 15.1 years. *AIRE* mutations were determined.

**Results:**

The prevalence of APS-1 was 2.6 cases/million (range 0.5–17 in different regions). At the onset 93% of patients presented with one or more components of the classical triad and 7% with other components. At the end of follow-up, 86.1% had CH, 77.2% AD, 74.7% CMC, 49.5% premature menopause, 29.7% autoimmune intestinal dysfunction, 27.8% autoimmune thyroid diseases, 25.9% autoimmune gastritis/pernicious anemia, 25.3% ectodermal dystrophy, 24% alopecia, 21.5% autoimmune hepatitis, 17% vitiligo, 13.3% cholelithiasis, 5.7% connective diseases, 4.4% asplenia, 2.5% celiac disease and 13.9% cancer. Overall, 991 diseases (6.3 diseases/patient) were found. Interferon-ω Abs (IFNωAbs) were positive in 91.1% of patients. Overall mortality was 14.6%. The *AIRE* mutation R139X was found in 21.3% of tested alleles, R257X in 11.8%, W78R in 11.4%, C322fsX372 in 8.8%, T16M in 6.2%, R203X in 4%, and A21V in 2.9%. Less frequent mutations were present in 12.9%, very rare in 9.6% while no mutations in 11% of the cases.

**Conclusions:**

In Italy, APS-1 is a rare disorder presenting with the three major manifestations and associated with different *AIRE* gene mutations. IFNωAbs are markers of APS-1 and other organ-specific autoantibodies are markers of clinical, subclinical or potential autoimmune conditions.

**Supplementary Information:**

The online version contains supplementary material available at 10.1007/s40618-021-01585-6.

## Introduction

Autoimmune Polyendocrine Syndrome type 1 (APS-1) (OMIM 240300), also termed Autoimmune Poly-Endocrine-Candidiasis-Ectodermal-Dystrophy (APECED) [1] or multiple autoimmune syndrome type 1 (MAS-1) [2, 3] is a rare disease with a mean prevalence of 10 cases per million inhabitants [4]. The prevalence varies and is higher in patients with consanguinity or in some particular populations [5]. The highest reported prevalence is in the Iranian Jewish population (1 case per 9000 individuals) [6], followed by Finland (1 case per 25,000 individuals) [1], Norway (1 case per 90,000 individuals) [7] Poland (1 case per 129,000 individuals) [8], and Ireland (1 case per 130,000 individuals) [9] with the lowest prevalence in France (1 case per 500,000 individuals) [10] and in Japan (1 case per 10 million individuals) [5, 11]. APS-1 is defined by the presence of at least two of the three major diseases: chronic mucocutaneous candidiasis (CMC), chronic hypoparathyroidism (CH) and Addison’s disease (AD) [12]. In addition, patients present with other autoimmune and non-autoimmune diseases and ectodermic dystrophy [1, 4, 12–14]. In European cohorts, one or more major manifestations presented at the onset in 80–90% of patients while a small proportion (5–20%) presented with other diseases [5, 13, 15, 16]. In contrast, in an American cohort, 40–80% of patients had non-major diseases at manifestation onset and the development of classical manifestations was delayed [17]. These observations have led to establishing new criteria for diagnosing APS-1 which recommend testing for interferon autoantibodies in patients presenting one of the following clinical manifestations: CMC, AD (occurring below 20 years of age), CH, premature ovarian failure (occurring below 30 years of age), enamel hypoplasia, periodic fever with rash, non-infectious keratitis or autoimmune hepatitis. If testing for interferon autoantibodies is not available it is recommended to proceed directly with *AIRE* gene analysis [4]. APS-1 is caused by autosomal recessive mutations of *AIRE* gene on chromosome 21 (21q22.3). *AIRE* gene plays an essential role in central tolerance. Mutations in the *AIRE* gene prevent the elimination of self-reactive T cells at central level and induce a Treg defect at peripheral levels [4, 18, 19]. This leads to the development of multiple autoimmune diseases at a young age [4, 20, 21]. The most frequent mutation is R257X on exon 6 detectable in 87% of Finnish patients although it was also found in other populations [4, 5, 15, 22, 23]. Mutations C322fsX372 or del13 in exon 8 are typical of Anglo-Saxon populations but can also occur in patients of different descent [4, 5, 22–24]. To date, more than 126 *AIRE* mutations have been identified [4, 5, 20]. Although APS-1 is very rare, 20 cohorts were described from 1998 to 2018 [6, 8–11, 15, 17, 20, 24–35] and a total of 568 patients from different populations have been assessed to date (Table 1 and Supplementary Figure 1). Several reports of Italian patients with APS-1 were published from 1974 to 2016 [22, 26, 36–53]. However, a complete analysis on all the patients with APS-1 in Italy has not been carried out so far. The aim of this study was to collect data on all the APS-1 patients living in Italy. In collaboration with the Italian Society of Endocrinology (SIE), the Italian Society of Pediatric Endocrinology and Diabetology (SIEDP), the Association of Medical Endocrinologists (AME) and the Italian Association of Patients with Addison’s disease (AIPAd), all the APS-1 patients who were diagnosed and followed up in different specialist endocrine centers in Italy have been recruited, their sera and/or DNA samples were collected and the first Italian national register of patients with APS-1 has been created.

**Table 1:** Main features of APS-1 presentation in patients from 20 different national cohorts published from 1992 to 2018

	[6] Iranian Jewish (1992)	[24] UK (1998)	[25] USA (1998)	[26] Italy (1998)	[27] Central-East Europe (2001)	[11] Japan (2002)	[28] Slovenia (2005)	[15] Finland (2006)	[8] Poland (2006)	[9] Ireland (2006)	[29] Slovakia (2008)	[10] France (2012)	[30] Hungary (2010)	[31] Saudi Arabia (2010)	[32] Northern Ireland (2012)	[20] Norway (2016)	[17] North/South America (2016)	[33] Russia (2017)	[34] India (2017)	[35] Brazil (2018)	Total or range
No of patients	23	12	20	41	27	7	12	91	14	31	4	19	7	20	8	52	35	112	23	13	568
F/M	1.1	0.8	1.5	2.4	n/a	0.75	0.3	1	1.8	1.4	1	0.6	0.75	1.2	0.6	0.85	1.5	1.3	0.9	3.3	0.3–3.3
Mortality (%)	n/a	28	n/a	4	n/a	n/a	n/a	29	n/a	10	n/a	10	14	n/a	0	28	n/a	8	26	23	0–29
Familial disease (%)	56	61	15	17.5	15	n/a	33	32	29	42	n/a	53	57	100*	50	53	n/a	13	53	25	13–57
CMC (%)	17	100	80	83	85	85	100	100	93	80	0	89	43	90	87	77	86	75	96	69	17–100
CH (%)	96	89	100	93	92	71	83	88	100	84	50	63	71	100	87	73	85	78	91	77	50–100
AD (%)	22	83	95	73	89	43	58	84	57	68	100	79	57	40	87	63	90	67	55	15	22–100
AITD (%)	4	5	25	10	26	n/a	25	31	n/a	6	50	5	29	15	n/a	19	22	13	23	63	4–63
POF (%)	25	37	8	43	n/a	n/a	0	50	3	68	50	71	n/a	9	33	33	38	48	60	23	0–71
DM-1 (%)	4	28	n/a	3	3	n/a	8	33	33	13	n/a	5	n/a	15	25	8	11	9	9	15	3–33
GHD (%)	n/a	n/a	n/a	7	7	n/a	58	5	7	6	n/a	5	14	5	12	n/a	n/a	n/a	n/a	39	5–58
AG/PA (%)	9	22	n/a	15	15	n/a	n/a	31	7	6	27	21	n/a	5	12	15	4	8	45	n/a	4–45
CD (%)	n/a	n/a	n/a	n/a	n/a	n/a	n/a	n/a	n/a	n/a	n/a	n/a	14	5	n/a	n/a	n/a	n/a	n/a	8	5–14
AIH (%)	n/a	17	25	20	15	n/a	8	18	43	19	n/a	11	14	15	12	4	14	11	n/a	31	4–43
AID (%)	n/a	28	5	15	18	n/a	25	22	14	13	n/a	26	n/a	n/a	n/a	23	54	25	27	8	5–54
K (%)	n/a	11	n/a	12	18	n/a	17	22	43	16	n/a	37	n/a	5	50	12	14	13	9	23	5–50
A (%)	13	22	40	37	37	n/a	33	39	21	19	25	53	n/a	45	12	31	7	34	6	16	6–53
V (%)	n/a	n/a	10	15	11	n/a	8	31	n/a	6	n/a	21	n/a	n/a	50	15	7	9	6	39	6–50
ND (%)	4	n/a	n/a	7	26	n/a	42	n/a	n/a	n/a	n/a	5	n/a	5	n/a	13	14	n/a	4	39	4–42
EH (%)	4	n/a	n/a	n/a	n/a	n/a	n/a	n/a	n/a	72	25	37	n/a	72	n/a	28	61	n/a	n/a	n/a	4–72
As (%)	17	n/a	n/a	11	n/a	n/a	n/a	19	n/a	n/a	n/a	5	n/a	n/a	n/a	16	n/a	2	5	n/a	2–19

*CMC* chronic mucocutaneous candidiasis; *CH* chronic hypoparathyroidism; *AD* Addison’s disease; *AITD* autoimmune thyroid diseases, *POF* premature ovarian failure; *DM-1* diabetes mellitus type 1; *GHD* GH deficiency; *AG/PA* autoimmune gastritis, pernicious anemia; *CD* celiac disease; *AIH*(autoimmune hepatitis; *AID* autoimmune intestinal disease; *K* keratoconjunctivitis; *A* alopecia; *V* vitiligo; *ND* nail dystrophy; *EH* enamel hypoplasia; *As* asplenia

^*^Included 7 families with multiple members affected, n/a (data not available). For studies in different populations/countries, the year of the study is shown in the brackets below and the respective reference number is shown in the brackets above the country

## Patients and methods

### Patients

One hundred and sixty-seven patients with APS-1 were enrolled into the study. Data on age, gender, manifestations, serum biochemistry and serum autoantibody profiles at onset and during a follow-up were collected. The diagnosis of the various diseases was performed according to the criteria previously published [3, 54] and summarized in the Table 2. Furthermore, *AIRE* gene mutations were analyzed. APS-1 was diagnosed using the classical clinical criteria defined in 1980 [12] or according to the new criteria [4]. The ethnic origin and for Italian patients the geographical region of provenance of their families were also recorded. The study was performed according to the principles of the Helsinki declaration. The patients gave their written consent to participate in this study. The study was approved by the ethical committee of the Azienda Ospedaliera-Universitaria in Padua, Italy (Ref. No 1299P and 1583P).

**Table 2:** Clinical manifestations, diagnostic criteria and autoantibody tests in APS-1 patients

Disease	Main clinical manifestations	Diagnostic tests	Mean age of onset (range)	Autoantibodies
Chronic mucocutaneous candidiasis	Chronic candida infection of mucosae, nails, esophagus	Clinical evaluation and culture	9.1 yrs (0.5–79)	IFNAbs, ILAbs
Chronic hypoparathyroidism	Paraesthesia, tetany, muscle cramps, Trousseau/Chvostek's signs	Calcium, PTH	11.1 yrs (0.5–74)	NALP-5Abs CaSRAbs (?)
Addison’s disease	Fatigue, hyperpigmentation, hypotension, weight loss, nausea	ACTH, cortisol, renin, ACTH test	16.3 yrs (2–76)	21-OHAbs, ACA
Premature ovarian failure	Primary or secondary amenorrhea, infertility	Estradiol, FSH, LH, anti-mullerian hormone	22.7 yrs (12–39)	StCA, 17αOHAbs, SCCAbs, 21-OHAbs
Testicular failure	Erectile dysfunction, infertility	FSH, LH, testosterone	Very rare	StCA, 17αOHAbs, SCCAbs, 21-OHAbs
Autoimmune thyroid diseases	Signs/symptoms of hypothyroidism or hyperthyroidism	TSH, FT4, thyroid ultrasound	18.6 yrs (3–64)	TPOAbs, TgAbs, TRAbs
Type 1 diabetes mellitus	Polyuria, polydipsia, weight loss	Glucose, c-peptide, HbA1c	18.1 yrs (1.5–39)	ICA; GADAbs, IA2Abs
Pituitary deficiency	Signs/symptoms of secondary hypothyroidism, hypogonadism, adrenal insufficiency, growth retardation	TSH, FT4, FSH, LH, estradiol, testosterone, ACTH, Cortisol, HGH, IGF-1	13.7 yrs (4–32)	Pituitary Abs, ECE-2Abs
Atrophic Gatritis/pernicious anemia	Dyspepsia, anemia	Blood count, Iron, vitamin B12, pepsinogen I, gastrin, gastric biopsy	23.0 yrs (4–56)	PCA, IFA
Autoimmune intestinal disease	Chronic diarrhea or costipation	Serotonin, gastric/duodenal biopsy	14.1 yrs (1–32)	TPHAbs, HDAbs, AADCAbs
Celiac disease	Intestinal and extraintestinal manifestations	Duodenal biopsy, total IgA	Variable	tTGAbs-IgA
Autoimmune hepatitis	Dyspepsia, fatigue	AST, ALT, γGT, bilirubin, liver ultrasound and biopsy	13 yrs	CYPIA2Abs, ActineAbs
Vitiligo	Areas of skin depigmentation	Clinical evaluation	17 yrs (3–62)	SOXAbs, MPCAbs
Alopecia	Local, total or universal hair loss	Clinical evaluation	13 yrs (2–40)	THAbs
Ectodermal dystrophy	Keratoconjunctivitis, enamel hypoplasia, nail dystrophy, tympanic calcifications, cataract, punctate nail defect	Clinical evaluation	10–16 yrs	IFNAbs
Asplenia	Susceptibility to bacterial infections	Howell–Jolly bodies, target cells on blood smears, abdominal CT or ultrasound	25 yrs	IFNAbs
Haematological diseases	Autoimmune anemia, thrombocytopenia, neutropenia	Blood count	Variable	IFNAbs
Hypokalemia with hypertension	Hypertension, muscle cramps	Na, K, Cl	Variable	IFNAbs
Pulmonary diseases	Pulmonary hypertension, bronchiolitis, bronchiectasis	Thorax CT	Variable	KCNRGAbs
Exocrine pancreas insufficiency	Diarrhea, malabsorption	Amylase, lipase	Variable	IFNAbs
Prostatitis	Fever, dysuria, stranguria	Prostate ultrasound	Variable	Tr-4Abs

### AIRE gene mutation

All 14 exons of the *AIRE* gene and their flanking exon-intron boundaries (GenBank accession no. Aj009610) were analyzed in 136 patients and in 170 non affected family members using previously described methods [22]. Data were analyzed for the total Italian cohort and also for groups of patients according to their regional origin.

### Autoantibodies

Patients’ sera were screened for interferon-ω autoantibodies (IFNωAbs) and for the range of organ and non-organ-specific autoantibodies relevant for various autoimmune diseases using methods described previously [26, 41, 55–62]. Autoantibody positivity confirmed the diagnosis in patients who presented with clinical autoimmune diseases. Autoantibody positive patients who did not display clinical signs/symptoms and/or had no biochemical markers of a subclinical disease were periodically followed up to assess the progression to the disease and to calculate the annual incidence of the disease.

### Statistical analysis

Comparison of female/male ratios, correlations between autoantibodies and the specific diseases, genotype and phenotype, geographic provenance and phenotype were analyzed by Chi-square test. Statistical significance was determined at *p* < 0.05. Survival analysis was performed using Kaplan–Meier analysis. Differences between subgroups were tested with Log-Rank and Cox regression.

## Results

### Epidemiology

Between 1965 and 2019, there were 167 patients with APS-1 living in Italy. Of these, 158 were Italians while 9 migrated to Italy from other countries. Out of the 158 Italian APS-1 patients, 103 (65.2%) were females and 55 males, with a female/male ratio of 1.9/1. Considering the size of the Italian population of 60,589,085 individuals [63], the prevalence of APS-1 could be estimated at about 2.6 cases/million, corresponding to one case per 384,615 individuals. The prevalence of APS-1 in different Italian regions or macro-areas was also estimated [63] and is summarized in Table 3. Furthermore, “hot spot areas” for APS-1 were identified in three Italian regions, for example, 12/28 of cases in Sardinia were found in the “Ogliastra” area, 11/24 of Veneto region cases in “Bassano del Grappa” and 12/15 of Apulia cases in “Salento”. The patients were followed up from diagnosis of APS-1 until the date of death or to the end of 2019. The mean follow-up period was 23.7 ± 15.1 years (range 1–67 years). The mean age of the patients at the entry and at the end of the follow-up period was 8.3 ± 11.8 years (range 0.5–76) and 32 ± 18 years (range 3–81 years), respectively.

**Table 3:** Prevalence of APS-1 in the Italian population overall and in different Italian geographic regions or macro-areas

Geographical origin of APS-1 patients	Italian population [62]	Number of Italian patients with APS-1	Prevalence of patients/million inhabitants
Italy (total cases)	60,589,085	158	2.6
Sardinia	1,653,135	28	17.0
North-East Italy	7,188,201	28	3.9
Veneto	4,907,529	24	4.9
Trentino-Alto Adige	1,062,860	3	2.8
Friuli Venezia Giulia	1,217,872	1	0.8
Sicily	5,056,641	20	3.9
Apulia	4,063,888	15	3.7
Campania	5,839,084	15	2.6
South Italy	2,535,493	8	3.2
Calabria	1,965,128	7	3.6
Basilicata	570,365	1	1.8
North-West Italy	15,976,639	14	0.9
Lombardy	10,018,806	6	0.6
Piedmont	4,392,526	7	1.6
Liguria	1,565,307	1	0.6
Central Italy	15,627,457	13	0.8
Lazio	5,898,124	6	1.0
Emilia-Romagna	4,448,841	4	0.9
Tuscany	3,742,437	2	0.5
Marche	1,538,055	1	0.6
Patients with parents originating from 2 regions		8	n.d
Patients with parents of unspecified regional origin		9	n.d

Data on populations were taken from http://www.comuni-italiani.it/regionip.html [62]

*n.d.* not determined

### APS-1 disease components at the onset and by the end of follow-up

At the onset of APS-1, 147/158 patients (93%) showed at least one component of the classical triad, while 11/158 (7%) presented with other conditions.

#### Chronic mucocutaneous candidiasis (CMC)

**Fig. 1:**
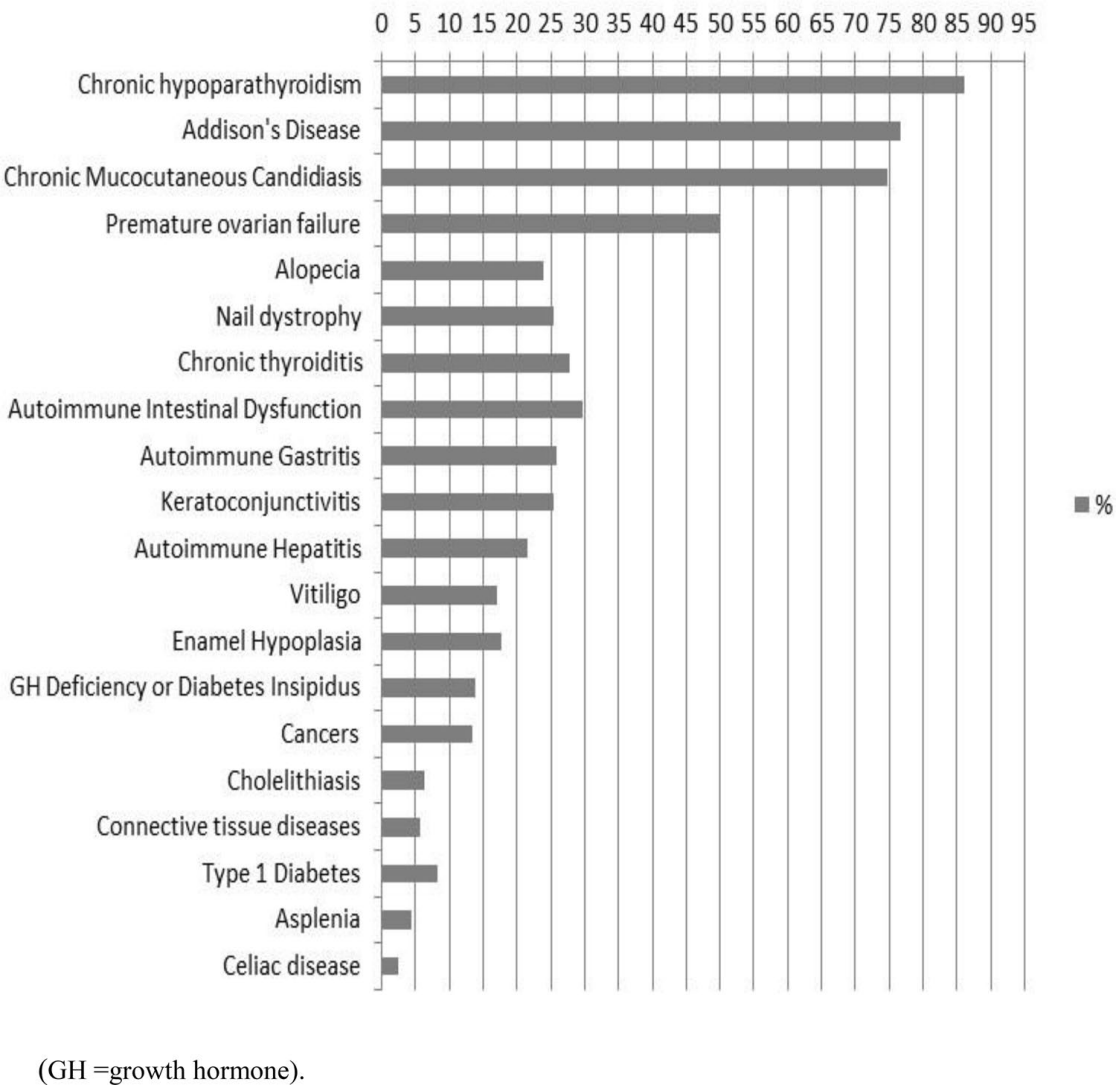
Clinical manifestations at the end of follow-up of APS-1 patients in Italy, presented as a percent of the total number of patients (*n* = 158). *GH* growth hormone

**Fig. 2:**
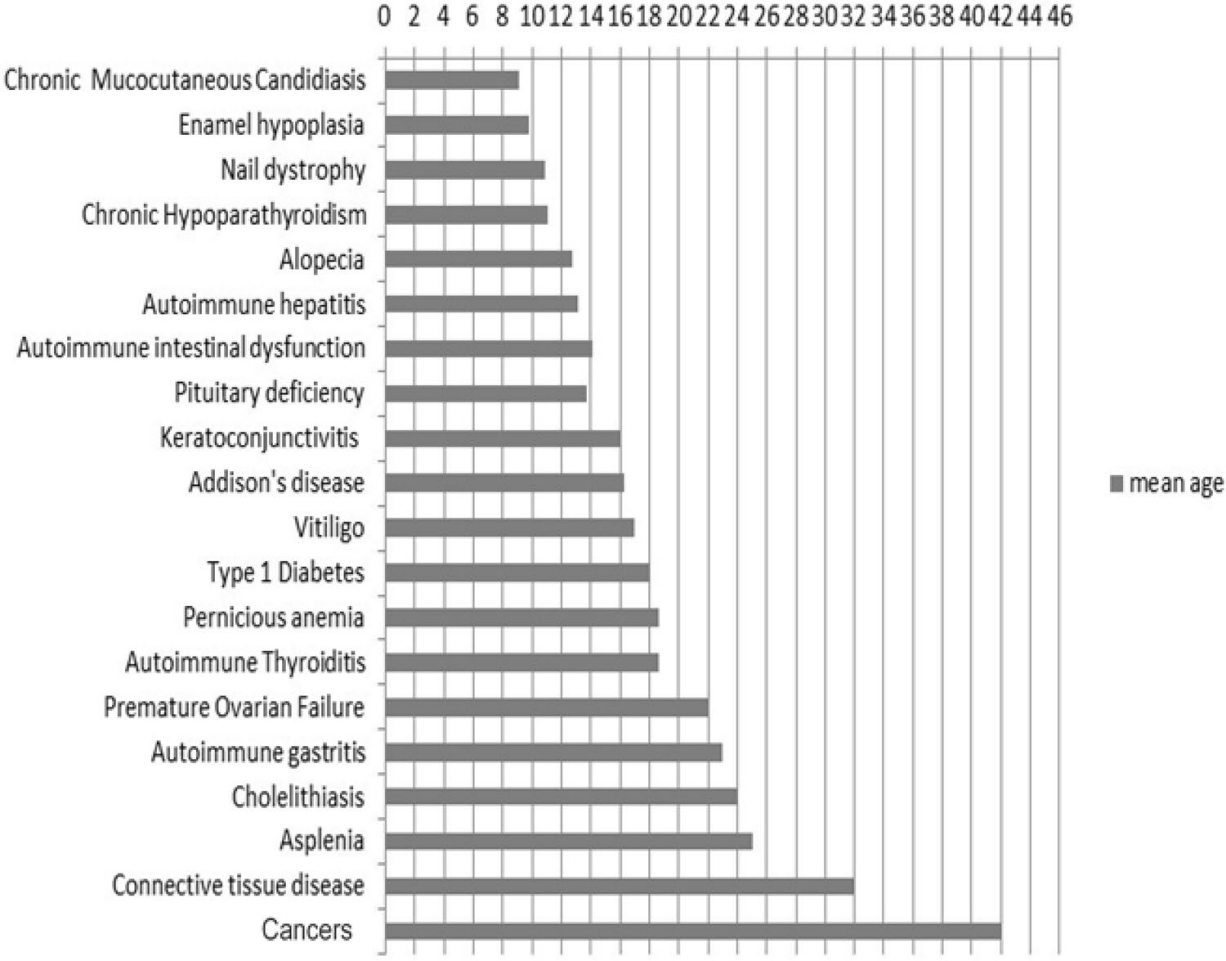
Mean age (in years) at the onset of different clinical manifestations in Italian patients with APS-1

**Fig. 3:**
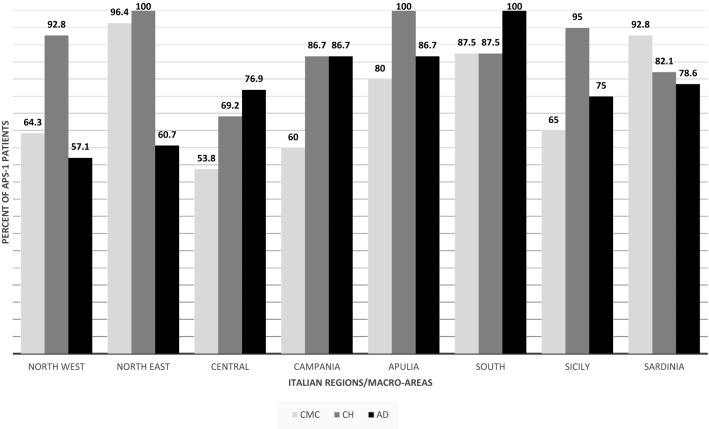
Occurrence of CMC, CH and AD in patients with APS-1 from various Italian regions or macro-areas

CMC was present at the onset of APS-1 in 81/158 patients (51.3%). In 60/158 (38%), CMC was isolated, while in 21/158 (13.3%), it manifested with other diseases. CMC presented at a mean age of 4.4 ± 6.4 years (range 0.5–36 years). By the end of follow-up, 118/158 patients (74.7%) were affected by CMC with a mean age at diagnosis of 9.1 ± 13.6 years (range 0.5–79 years) (Figs. 1, 2). In detail, 12/118 patients (10.2%) developed CMC by the age of 1 year, 26 (22%) by the age of 2 years, 15 (12.7%) between 2 and 3 years, 22 (18.6%) between 4 and 6 years, 13 (11%) between 7 and 10 years, and 25 (21.2%) after 10 years of age. For five patients (4.2%), the age when CMC was diagnosed was not available. The occurrence of CMC in different geographical regions in Italy is summarized in Fig. 3. During follow-up, six patients developed dysphagia and esophageal stenosis requiring periodical endoscopy for pneumatic dilatations.

#### Chronic hypoparathyroidism (CH)

CH was the second most common presentation and the first endocrine disorder at onset of APS-1 in 55/158 (34.8%) patients. 34/158 (21.5%) patients manifested with isolated CH, while in 21/158, CH was associated with other diseases (13.3%). CH developed at a mean age of 8.4 ± 10.2 years (range 0.5–56 years). By the end of follow-up, CH was the most frequent disease affecting 136/158 patients (86.1%) and presenting at a mean age of 11.1 ± 12.1 years (range 0.5–74 years) (Figs. 1, 2). In all, 6/136 patients (4.4%) developed CH in the first 2 years of life, 23 (17%) between 2 and 3 years of age, 41 (30.1%) between 4 and 6 years, 23 (16.9%) between 7 and 10 years, 19 (14%) between 11 and 20 years, and 20 (14.7%) after 20 years of age. For 4 patients (2.9%), the age at presentation of CH was unknown. Figure 3 shows the presentation of CH in Italian regions. NALP-5Abs were measured in a group of 41 patients with a mean disease duration of 19 years (range 1–30 years) and 18 (44%) tested positive.

#### Addison’s disease (AD)

AD developed in 27/158 (17%) patients at the onset of APS-1, in 10/158 (6.3%), it was isolated and in 17/158 (10.7%), associated with other diseases. AD developed at a mean age of 18.8 ± 18.6 years (range 2–76 years). By the end of the follow-up, 122/158 patients (77.2%) manifested AD at a mean age of 16.3 ± 14.1 years (range 2–76) (Figs. 1, 2). In 9/121 patients (7.4%), AD was diagnosed by 3 years of age, in 18 (14.9%) between 4 and 6 years, in 18 (14.9%) between 7 and 8 years, in 17 (14%) between 9 and 10 years, in 26 (21.5%) between 11 and 20 years, in 13 (10.7%) between 21 and 30 years, and in 18 (14.9%) after 30 years. The age at AD onset was not available for three patients. The presentation of AD in APS-1 patients from different Italian regions is summarized in Fig. 3. Adrenal cortex autoantibodies (ACA) were found in 84/104 (80.8%) and 21-OH autoantibodies (21-OHAbs) in 75/97 patients (77.3%) with different AD duration. Out of 53 patients with APS-1 without AD 29 (54.7%) were positive for ACA and/or 21-OHAbs. Adrenal cortex autoantibody-positive patients were followed up for a mean period of 10 years (range 1–30 years) and 25/29 (86.2%) developed AD with an overall incidence of AD of 27% per year.

### Prevalence of other endocrine and non-endocrine autoimmune diseases at the onset of APS-1 and by the end of follow-up

#### Hypergonadotropic hypogonadism (HH)

##### Females

HH, also known as premature ovarian failure (POF) in females, was found in one 30-year-old patient at the onset of APS-1. By the end of the follow-up, 51/103 females (49.5%) developed POF at a mean age of 22 ± 7.2 (range 12–39 years) (Figs. 1, 2). In 43 patients (84%), POF developed as secondary and in 8 (16%) as primary amenorrhea. Two patients had Turner’s syndrome. Steroid-producing cell autoantibodies (StCA) and/or 17αOH autoantibodies (17αOHAbs) and/or side-chain cleavage autoantibodies (SCCAbs) were positive in 35/41 (85.4%) patients with POF and in 24/42 (57.1%) without POF. During follow-up, 10/24 (41.7%) autoantibody-positive patients progressed to POF, with an overall incidence of 5.6% per year.

##### Males

None of the males with APS-1 showed HH at the onset of APS-1. StCA and/or 17αOHAbs and/or SCCAbs were positive in 23/38 (60.5%) tested without HH. During a 7.6-year follow-up only one of the autoantibody-positive patients developed HH at the age of 16 years (after pubertal development).

#### Autoimmune thyroid disease (AITD)

Nine of 158 patients (5.7%) were diagnosed with AITD at the onset of APS-1. In 4 patients (2.5%), AITD was isolated, while in 5 (3.2%), associated with other diseases and developed at a mean age of 14.8 ± 6.2 years (range 3–37 years). By the end of follow-up, 44/158 patients (27.8%) developed AITD at a mean age of 18.6 ± 13.4 years (range 3–64 years) (Figs. 1, 2). The majority, 41/44 (93.2%) presented with chronic thyroiditis (CT) while 3/44 (6.8%) with Graves’ disease (GD). At entry to follow-up, 10/30 (33.3%) patients tested positive for thyroid autoantibodies had a subclinical hypothyroidism while 20/30 (66.7%) had normal thyroid function. During follow-up, 6/20 thyroid autoantibody-positive patients (30%) developed hypothyroidism, with an annual incidence of 4.5%.

#### Type 1 diabetes mellitus (DM-1)

Isolated DM-1 developed at the onset of APS-1 in 2/158 patients (1.3%) (at 18 months and 39 years of age, respectively). By the end of the follow-up, 13/158 (8.2%) patients developed DM-1 at a mean age of 18.1 ± 12.6 years (range 18 months–39 years) (Figs. 1, 2). At the onset of DM-1, all affected patients were positive for glutamic acid decarboxylase autoantibodies (GADAbs) and/or islet cell autoantibodies (ICA). Furthermore, 18/64 patients (28.1%) without DM-1 were found positive for ICA and/or GADAbs and during follow-up, the annual incidence of clinical DM-1 was 1.1%.

#### Pituitary insufficiency

None of the 158 patients presented with pituitary insufficiency at the onset of APS-1. By the end of follow-up, 22/158 (13.9%) developed pituitary insufficiency at a mean age of 13.7 ± 8.1 years (range 4–32 years) (Figs. 1, 2). Of these, 15 patients (68.2%) had isolated growth hormone (GH) deficiency, 3 (13.6%) diabetes insipidus, 1 (4.5%) hypogonadotropic hypogonadism and 3 (13.6%) had GH deficiency associated with hypogonadotropic hypogonadism. One out of four patients tested for pituitary autoantibodies was positive.

#### Autoimmune gastritis (AG)/pernicious anemia (PA)

At the onset of APS-1, AG was observed in one 4-year-old patient. However, 41/158 (25.9%) patients developed AG at a mean age of 23 ± 13 years (range 4–56) by the end of follow-up (Figs. 1, 2). Macrocytic anemia suggesting PA was detected in 21/41 patients with AG and was diagnosed at a mean age of 18.5 years (Fig. 2). Out of 38 patients with AG tested for parietal cell autoantibodies (PCA), 27 (71%) were positive. Intrinsic factor autoantibodies (IFA) were tested in 21 patients with PA and were positive in 13 (62%).

#### Vitiligo

At the onset of APS-1, 3/158 (2%) patients had vitiligo (isolated in one, associated with other diseases in two) that developed at a mean age of 4 ± 1.7 years. However, by the end of follow-up, vitiligo developed in 27 (17.1%) subjects at a mean age of 17 ± 15 years (range 3–62 years) (Figs. 1, 2). Melanin-producing cell autoantibodies (MPCAbs) were found in 10/18 tested patients with vitiligo and in one without vitiligo who developed vitiligo 10 years later.

#### Alopecia

Only 3/158 (2%) patients at the onset of APS-1 had alopecia which presented in association with other diseases at 3 years of age in all three subjects. In all, 38/158 (24%) patients developed alopecia by the end of follow-up at a mean age of 13 ± 10 years (range 2–40 years) (Figs. 1, 2).

#### Celiac disease (CD)

None of the 158 patients had CD at the onset of APS-1 while 4/158 (2.5%) tissue transglutaminase autoantibodies-IgA (tTgAbs-IgA)-positive patients developed CD by the end of follow-up (Fig. 1).

#### Autoimmune intestinal dysfunction (AID)

Two APS-1 patients (1.3%) manifested diarrhea or obstinate constipation, at the onset of APS-1; one had isolated AID diagnosed at 1 year of age, while the other presented at 32 years of age and had AID associated with other conditions. During the follow-up, 47/158 patients (29.7%) developed AID at a mean age of 14.1 ± 8.3 years (range 1–32 years) (Figs. 1, 2). TPHAbs were positive in 31/37 (83.8%) and AADC in 36/37 (97.3%) patients with diarrhea or obstinate constipation. Gastric and duodenal biopsies in eight autoantibody-positive patients with diarrhea or obstinate constipation showed the presence of CD3^+^/CD8^+^ intraepithelial lymphocytes with a reduced number of enterochromaffin cells. The mean serum levels of serotonin in these patients were low of 0.16 ± 0.14 μM/L (normal range: 0.28–1.7 μM/L). The mean serotonin levels in other eight TPHAb-positive patients without diarrhea or obstinate constipation were within the lower levels of normal range at 0.58 ± 0.41 μM/L suggesting a subclinical AID.

#### Autoimmune hepatitis (AIH)

AIH was diagnosed at the onset of APS-1 in 4/158 patients (2.5%) and was isolated in 3 patients while associated with other manifestations in one. The mean age at diagnosis was 2.8 ± 2.2 years. By the end of follow-up, 34 patients (21.5%) developed AIH at a mean age of 13.1 ± 9.2 years (range 1–32 years) (Figs. 1, 2). Liver–kidney microsomal autoantibodies (LKMAbs) were positive in 13/24 patients (66.6%) with AIH, while 3/7 patients (43%) were positive for smooth muscle antibodies. The severity of the disease varied with some subjects having moderate to high levels of liver enzymes and responding to immunosuppression therapy while some (*n *= 4) progressing to acute fulminant hepatitis causing death.

#### Autoimmune connective disease (ACD)

None of the patients had ACD at the onset of APS-1. However, during follow-up, 9/158 (5.7%) developed ACD at a mean age of 32 ± 12 years (range 10–47 years) (Figs. 1, 2). Six patients had Sjogren’s syndrome, two rheumatoid arthritis, and one systemic scleroderma.

#### Spleen atrophy (SA)

None of the patients had SA at the onset of APS-1 while during follow-up, SA was detected in 7/158 patients (4.4%) at a mean age of 25 ± 10 years (range 5–33 years) (Figs. 1, 2).

#### Prevalence of other non-autoimmune diseases at the onset and by the end of follow-up

##### Ectodermal dystrophy

Keratoconjunctivitis (KC) was observed in one patient at the onset of APS-1 and by the end of follow-up in 40/158 patients (25.3%) at a mean age of 16 ± 8 years (range 2–36 years) (Figs. 1, 2). In addition, by the end of follow-up, enamel hypoplasia (EH) was noted in 28/158 patients (17.7%) at a mean age of 9.8 ± 3 years (range 6–18 years) (Figs. 1, 2) while 5/158 (3.2%) patients had nail dystrophy (ND) (2 isolated and 3 associated with other disorders) at diagnosis of APS-1 at a mean age of 4.4 ± 2.5 years (range 0.5–8 years). By the end of follow-up, ND was present in 40/158 patients (25.3%) and developed at a mean age of 10.9 ± 6.7 years (range 0.5–22 years) (Figs. 1, 2).

##### Cholelithiasis (Ch)

Ch was diagnosed at the onset of APS-1 in one 23-year-old patient. During the follow-up, 11/158 patients (7%) developed Ch at a mean age of 24 ± 8 years (range 11–27 years) (Figs. 1, 2).

##### Cancer

During follow-up, 23/158 patients (14.5%) developed 25 cancers at a mean age of 42 ± 9.6 years (range 24–68 years) (Figs. 1, 2). Of these, 7 patients had squamous cell carcinoma (SCC) of the oral mucosa, 1 had cancer of the tongue with local metastasis, 1 had cancer of the tongue and underwent tongue reconstructive surgery including quadriceps transplant, 1 patient with SCC of the tongue subsequently had peritoneal dissemination of gastric adenocarcinoma, 3 had esophageal cancer, 2 had gastric adenocarcinoma, 3 had large granular lymphocyte leukemia (LGLL) which in 1 patient was associated with pure red cell aplasia, 1 had a colorectal and a transverse colon adenocarcinoma, 1 had chronic lymphocytic leukemia, 1 had HIV-related Kaposi’s sarcoma, 1 had melanoma and 1 had lip carcinoma.

##### Other very rare diseases

Some of the patients in this study presented with other very rare diseases such as exocrine pancreas insufficiency, tubulointerstitial nephritis, hypopotassemia with apparent mineralocorticoid excess or sensitivity, deficit of IgA, metaphyseal dysplasia, vasculitis, autoimmune hemolytic anemia, cutaneous rash with fever, ocular myasthenia, obstructive pulmonary disease positive for potassium channel regulator autoantibodies (KCNRGAbs), psoriasis, pure red cell aplasia, posterior reversible encephalopathy syndrome, autoimmune demyelinating disease, cystic fibrosis, retinitis pigmentosa, chronic urticaria, lichen ruber planus, polyneuropathy, epilepsy, Hirschsprung’s disease, renal agenesia, toxic epidermal necrolysis, HCV and autoimmune myocarditis.

##### Overall diseases in patients with APS-1 at the end of follow-up

**Fig. 4:**
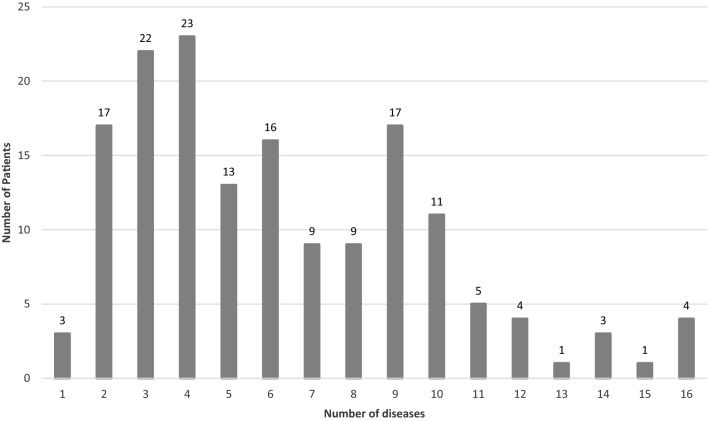
Number of APS-1 patients in the Italian population who presented with 1–16 associated diseases

By the end of the mean 23.7 ± 15.1 years (range 1–67 years) of follow-up, 991 diseases were documented in the Italian cohort of APS-1 patients with a mean of 6.3 per patient, ranging widely from 1 to 16 diseases/patient (Fig. 4).

##### Mortality at the end of follow-up

During the follow-up, 23/158 (14.6%) patients died at a mean age of 35.8 ± 21.3 years (range 5–78), 8/23 (34.8%) from cancers (2 from metastatic SCC of the oral mucosa, 3 gastric adenocarcinoma, 2 esophageal adenocarcinoma and 1 Kaposi’s sarcoma) and 13/23 (56.5%) from other diseases (4 fulminant AIH, 1 disseminated candidiasis, 1 cachexia, 1 adrenal crisis, 1 adrenal crisis during influenza with gastroenteritis, 1 brain hemorrhage, 1 pulmonary insufficiency, 1 Lyell’s syndrome, 1 kidney insufficiency, and 1 cerebral stroke). In two patients (8.6%), the cause of death was not defined. Mortality was significantly higher compared to the matched Italian population (*p *< 0.0001) [64] and greatly varied among patients from different geographical regions. In particular, during follow-up, 11/28 (39.3%) patients died in North-East regions, 4/20 (20%) in Apulia, 1/8 (12.5%) in the South, 3/28 (10.7%) in Sardinia, 2/20 (10%) in Sicily, 1/15 (6.7%) in Campania, and 1/44 (2.3%) in the other regions. Six patients reported a family history of an unexplained death of a sibling at a very young age.

#### Prevalence of IFNωAbs in patients with APS-1

IFNωAbs were positive in 103/113 (91.2%) patients tested. However, the 10 IFNωAb-negative patients were tested after a mean period of 25 years from the onset of the disease.

#### AIRE gene mutations

**Fig. 5:**
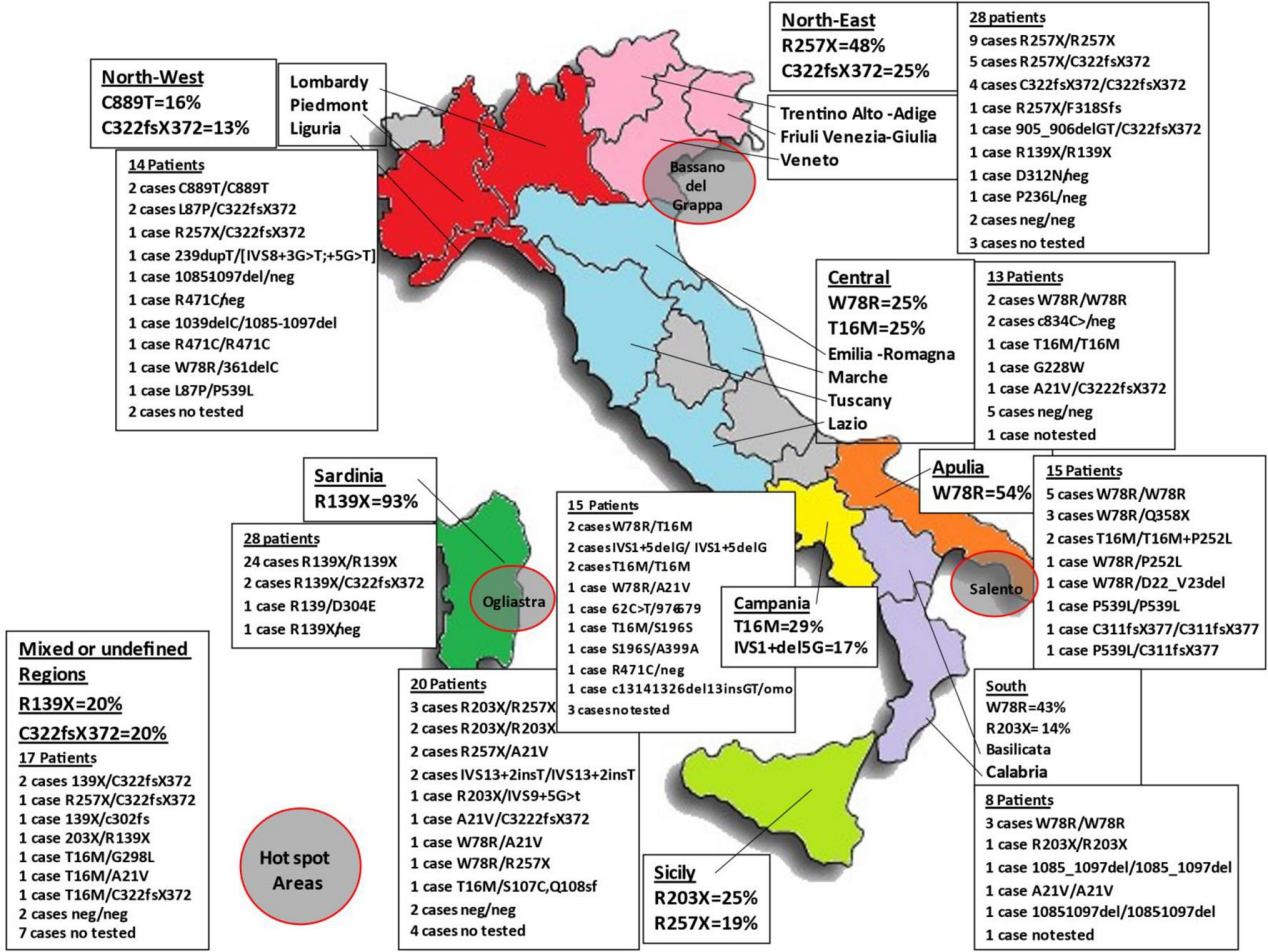
*AIRE* gene mutations in APS-1 patients in different macro-areas or regions of Italy

In all, the 158 Italian patients were from 138 families, of which 120 families (87%) had 1 affected member, while 18 families (13%) had more than 1 affected member; 2 in 17 families and in 1 family, 3/6 sisters were affected. Consanguinity between parents was found in 7/138 families (5%). *AIRE* gene mutations were investigated in 272 alleles from 136 patients. In 22 patients, *AIRE* gene screening was not carried out due to the lack of consent or to the unavailability of samples. The results of the *AIRE* gene mutations are summarized in Table 3. Overall, 116/136 patients (85.3%) had two homozygous or combined heterozygous mutations, 8 (5.9%) had 1 mutation, 11 (8%) had no mutations and 1 (0.7%) had 3 mutations. One of the eight patients with only one mutation had a dominant mutation (G228W) reported previously (41). The distribution of the *AIRE* gene mutations in relation to nine Italian geographical regions or macro-areas from which the families originated is summarized in Fig. 5 and supplementary Table 1.

The most frequent mutation was R139X detected in 58/272 alleles (21.3%) (Table 4). This mutation was almost exclusive 52/56 (93%) alleles in Sardinian patients (Fig. 5 and Supplementary Table 1). R139X was inversely correlated to AITD (*p* = 0.02). R257X was detected in 32/272 alleles (11.8%) (Table 3) and was present predominantly in patients from North-East regions (48%) and from Sicily (19%) (Fig. 5 and Supplementary Table 1). This mutation was associated with AG (*p* = 0.03), PA (*p* = 0.04), AID (*p* = 0.03), number of presented diseases (*p* = 0.04) and mortality (*p* = 0.03). W78R was found in 31/272 alleles (11.4%) (Table 3), mainly in patients from Apulia (54%), southern (43%) and central regions (25%) (Fig. 5 and Supplementary Table 1). W78R correlated with AD (*p* = 0.02), AITD (*p* < 0.01), KC (*p* < 0.01), and EH (*p* < 0.01). The less prevalent C322fsX372 mutation was detected in 24/272 alleles (8.8%) (Table 4), and present in patients from the North-East (25%), North-West macro-areas (13%) and mixed regions (19%) (Fig. 5 and Supplementary Table 1). There was no correlation between genotype and phenotype for this mutation. Further, 17/272 alleles (6.2%) carried T16M mutation (Table 3) which was found in patients from Campania (29%) and central regions (25%) and (Fig. 5 and Supplementary Table 1) and showed correlation with CMC (*p* = 0.03). R203X was detected in 11/272 alleles (4%) of Italian patients (Table 3), was present in Sicily (25%) and in the south regions (14%) (Fig. 5 and Supplementary Table 1) and was significantly associated with AITD (*p* = 0.03) and inversely associated with cholelithiasis (*p* = 0.01). A21V was detected in 8/272 alleles (2.9%) and found in Sicily (13%) (Table 3). Of rare mutations, 4/272 alleles (1.5%) carried 1085-1097-del, IVS13+2insT, IVS1+5delG and P539L, respectively, L87P was carried in 3/272 alleles (1.1%), and S196S, C311fsX377, c.965_977del13, C1314-1326del13/insGT, and c834 were carried in 2 alleles (0.7%), respectively (Table 3). Very rare mutations were found in other 26/272 (9.6%) alleles. One patient from Sicily had three mutations T16M/S107C, Q108sf (Fig. 5 and Supplementary Table 1) of which S107C and Q108fs are mutations located in exon 3 previously described [51, 52]. A patient from Campania had a homozygous mutation c1314-1326del13insGT described previously [46].

**Table 4:** *AIRE* gene mutations in the Italian population with APS-1

*AIRE* gene mutations	Alleles affected (%)
R139X	58/272 (21.3%)
R257X	32/272 (11.8%)
W78R	31/272 (11.4%)
C322fsX372	24/272 (8.8%)
T16M	17/272 (6.2%)
R203X	11/272 (4.0%)
A21V	8/272 (2.9%)
Less frequent	35/272 (12.9%)
R471C	4/272 (1.5%)
1085-1097del	4/272 (1.5%)
IVS13 + 2insT	4/272 (1.5%)
IVS1 + 5delG	4/272 (1.5%)
P539L	4/272 (1.5%)
C889T	4/272 (1.5%)
L87P	3/272 (1.1%)
S196S	2/272 (0.7%)
C311fsX377	2/272 (0.7%)
C1314-1326del/insGT	2/272 (0.7%)
C834C > G	2/272 (0.7%)
Very rare	26/272 (9.6%)
No mutations on both alleles in 11 patients and no mutation in 1 allele in other 8 cases	30/272 (10.7%)

Two pairs of siblings who carried the same homozygous mutation presented with distinctly different phenotypes. One pair of a 17-year-old male and 15-year-old female from Campania both had homozygous IVS1+5delG mutation and while the male presented 16 different diseases, the female was affected by only 4 conditions. A different pair from Calabria carried homozygous W78R mutation and a 22-year-old brother presented with 5 different diseases while his 15-year-old sister had 9 different diseases.

## Discussion

Our study describes 158 well-characterized Italian patients with APS-1 who represent the largest one nation cohort of patients with APS-1 published to date. Previously large cohorts of 91 Finnish [15] or 112 Russian patients have been reported [33]. In all 568, APS-1 patients from 20 different cohorts have been described previously (Table 1 and supplementary Figure 1) and with addition of 158 patients from our study the total number of well-characterized patient cohorts with APS-1 has reached 726. In addition to the cohort studies, there are a number of case reports on the individual or familial presentations of APS-1 in different countries. Therefore, the overall number of APS-1 patients worldwide could be estimated at approximately 1000 individuals. In our study, patients were recruited from specialist endocrine centers across Italy allowing for the first time an estimate of the prevalence of APS-1 in Italy at 2.6 cases/million, i.e., approximately 1 case per 384,615 individuals. Although the number of patients enrolled was relatively high, the prevalence of APS-1 in Italy is lower than in Iranian Jews, Finland, Norway, Poland and Ireland [1, 5–11, 15, 20] while higher than in France and Japan. Our study showed large variations in the prevalence of APS-1 in different regions of Italy. For example, the highest prevalence was noted in Sardinia with 17 cases/million (1 case per 58,823 inhabitants), followed by Veneto with 4.7, Sicily with 3.9 and Apulia with 3.7 cases/million, respectively. In contrast, lower prevalence (0.6–0.5 cases/million) was observed in the other regions (Table 2). Furthermore, three “hot spot areas” for APS-1 were identified in Italy (Fig. 5), and in those areas *AIRE* gene screening in the healthy population would be helpful in detecting asymptomatic carriers who could then be offered appropriate genetic counseling.

In this study, more than 93% of patients presented with one of the classical triad diseases at onset while 7% manifested with other diseases. A similar clinical presentation has been described in European populations [5, 15]. However, in the cohorts of American patients [17, 35], non-major component diseases were noted at APS-1 onset in 40–80% of the cases.

In the majority of our Italian patients who presented with the classical triad at onset, the first most frequently manifesting disease was CMC followed by CH and then AD. However, by the end of a long follow-up, the most frequent disease was CH diagnosed in 86% of the patients at a mean age of 11 years, followed by AD in 77% of the patients at a mean age of 16.3 years and CMC in 76% of the patients which presented at a younger mean age of 9.1 years. The highest prevalence of CMC, CH and AD was in patients from the South and Apulia and the lowest from the central regions (Fig. 3). These differences may be related to the differences in genetic background of populations in these regions. Subsequent to initial presentation of the three major manifestations of APS-1, many other autoimmune and non-autoimmune diseases developed throughout patients’ lives. APS-1 is a most complex and puzzling autoimmune syndrome due to the great variety and severity of autoimmune and non-autoimmune comorbidities. In our cohort, patients manifested in total 991 diseases with an average of 6.3 conditions per patient. However, 4 exceptional patients in our study developed up to 16 diseases per patient representing the highest co-morbidity in APS-1 reported to date [19].

IFNωAbs were found to be the best serological marker of APS-1 regardless of the comorbidities and the duration of the syndrome as previously described [4, 20, 33–35, 48]. This confirms that IFNωAbs are a diagnostic marker for APS-1 and can be particularly helpful in early diagnosis in patients presenting with a single manifestation of APS-1 [4].

Patients in our study developed cancers at a relatively young age of which approximately half were SCC of the mouth, tongue, esophagus, probably related to the CMC or gastric adenocarcinoma probably related to AG and this is consistent with previous reports [65]. In the case of other cancers, LGLL is a monoclonal lymphoproliferative disease characterized by persistent and indolent lymphocytosis described in only 0.2 and 0.7 cases/million in the USA and Netherlands, respectively [64], and associated with autoimmune diseases (rheumatoid arthritis, Sjogren’s syndrome, autoimmune cytopenia, hemolytic anemia, or thrombocytopenia) [66, 67]. In our study, LGLL was found in 3/158 (1.9%) of APS-1 patients and this represent the highest frequency of this disorder in an autoimmune syndrome. Only two case reports of LGLL in APS-1 were published so far [68, 69]. Finally, one exceptional case developed two adenocarcinomas of the colon not previously reported in this syndrome.

The mortality in APS-1 patients was significantly higher compared to the general Italian population [64] in agreement with previous reports on European [20, 70–72] and non-European cohorts [34, 35]. Mortality was highest in the North-East regions compared to the other regions in Italy. In approximately one-third of our patients, death was caused by malignancies while in the other two-third causes were adrenal crises, fulminant hepatitis, or generalized candidiasis.

In addition to IFNωAbs, we have also assessed the value of other serological markers of autoimmune diseases. We confirmed the role of NALP-5Abs as markers of CH; however, this test is not widely available outside research laboratories [61]. ACA and/or 21-OHAbs showed excellent diagnostic sensitivity and were detected in the great majority of APS-1 patients with AD within 2 years of onset. Furthermore, they were also found in approximately half of APS-1 patients without AD and conferred a high risk (86.2%) of progression to AD in APS-1 compared to a low risk (25%) in patients with other types of APS [73]. As AD in APS-1 tends to develop after CMC and/or CH, it would seem appropriate to test for ACA and/or 21-OHAbs in all patients with CMC and/or CH. This strategy would alert physicians to the high probability of progression to clinical AD for patients positive for these autoantibodies and would be helpful in preventing unexpected life-threatening adrenal crisis events [74].

Nearly, half of the females with APS-1 in our cohort developed POF (Fig. 1). This is in agreement with previous cohort studies where POF was among the most frequent of the non-classical components of APS-1 (Table 1 and supplementary Figure 1). In our study, the great majority of the patients with POF were positive for StCA and/or 17αOHAbs and/or SCCAbs which are considered diagnostic for autoimmune oophoritis without the need for ovarian biopsy or further genetic investigations [75–77]. Furthermore, approximately half of the young females with APS-1 who had normal menses and were positive for StCA and/or 17αOHAbs and/or SCCAbs developed POF during follow-up with an annual incidence of 5.6%. This confirmed that these autoantibodies are also good predictive markers of POF [77]. In addition, StCA, and/or 17αOHAbs and/or SCCAbs were positive in 60% of the males with APS-1 of whom none had HH at the time of testing and during the follow-up only one patient developed HH. Consequently, in this study, positivity for autoantibodies to steroid-producing cells/steroidogenic enzymes was associated with a very different risk of HH in females and males in agreement with previous studies [77, 78].

Almost one-third of APS-1 patients had AITD which presented in most cases as CT and only exceptionally as GD consistent with previous reports [15]. Some patients with normal thyroid function were positive for thyroid autoantibodies at entry into the study and during the follow-up, developed hypothyroidism with an annual incidence of 4.5%. This would suggest that thyroid autoantibodies could be considered as markers of progressing to thyroid dysfunction also in APS-1.

During the follow-up, a third of the patients with APS-1 manifested diarrhea or constipation and the great majority of them were positive for TPHAbs and/or AADCAbs. These Abs correlated with lymphocytic infiltration of the gastric and duodenal mucosa, reduced number of enterochromaffin cells and with the low serum serotonin levels consistent with AID. Our study also confirmed that TPHAbs and/or AADCAbs are markers of a subclinical AID as reported previously [79, 80].

In cohorts described previously to date, 3–33% of patients with APS-1 had DM-1 (Table 1 and supplementary Figure 1). In our cohort, DM-1 was found in 8% of patients who were all positive for diabetes-associated autoantibodies. This represents one of the lowest co-occurrences of DM-1 and APS-1 reported thus far. Although 28% of our APS-1 patients without DM-1 were positive for ICA and/or GADAbs the annual risk of developing clinical DM-1 at the end of follow-up was very low (1%) compared to patients with other autoimmune diseases [81] or to healthy children [82]. This difference risk may be related to the absence of specific HLA genotypes predisposing to DM-1 in our APS-1 patients. In addition, GADAbs in patients with APS-1 may recognize different epitopes compared to those recognized by patients with DM-1 without APS-1 [83].

AG was observed in a quarter of our patients and a half of these manifested with PA at a very young age although PA usually manifests in elderly individuals. In addition, one-fifth of the APS-1 patients had AIH occurring at a mean age of 13 years. The majority of our patients were responsive to immunosuppression therapy but four patients died from fulminant hepatitis. CD has been reported in 5–14% of APS-1 patients in different cohorts (Table 1 and Supplementary Figure 1) while in our study, only 3% of patients were affected by CD which may be related to the absence of HLA genes predisposing to CD in our population.

Our study shows that patients with APS-1 exhibit a wide range of specific autoimmune disorders some of which are rare. Autoantibody tests for these rare conditions are often unavailable in routine diagnostic laboratories and it would be appropriate that these should be provided by specialized, designated reference laboratories in different countries to assist in a complete diagnostic work-up of APS-1.

In this study, 13% of families had at least one other family member with APS-1 and this is one of the highest prevalence of the same autoimmune syndrome in non-twin relatives. It is consistent with the high genetic risk for APS-1 associated with *AIRE* gene mutations. Consanguinity of parents was found in 5% of families in our study in contrast to earlier reports on different populations [6, 31].

Our cohort of patients displayed different *AIRE* gene mutations some of which were clustered in different geographical regions. This differs from the Finnish, Iranian Jewish or other populations where one specific mutation was predominantly found in the great majority of patients [6, 15]. In Italy, the most prevalent mutation was R139X detected in 21.5% of the alleles overall; however, it was predominant in Sardinia being present in 95% of patients. The next most prevalent mutation was R257X found in 11.8% of alleles overall while being the most frequent in Veneto and North-East areas. R257X mutation is the most common among the Finnish APS-1 population [15]. In our cohort, W78R was found in 11.5% of the alleles overall and it was carried by most of the patients from Apulia where it was first detected [42] and in the south of Italy. C322fsX372 was found in 8.1% of APS-1 patients typically in the Veneto and North-East populations. This is a common mutation in patients with an Anglo-Saxon background [21]. In our study, we have identified three novel mutations: S107C and Q108fs on exon 3 in patients from Sicily and c1314-1326del13insGT in a patient from Campania. In our study, 8 patients had only one allelic mutation as reported in other populations [33]. Seven of these had one non-dominant mutation while one patient from central Italy had a dominant mutation G228W described previously [41, 84]. The only other dominant mutation associated with APS-1 reported to date was p.C311Y (p932G>A) found in a patient from North Africa [85]. The relatively wide variations in *AIRE* gene mutations among Italian APS-1 patients may reflect the heterogeneity of the founder genes derived from different migrant populations passing through or settling and living in Italy over time. Furthermore, this study found that some of the mutations were typical for specific geographical regions of Italy. Therefore, recording a detailed family history is essential for patients being tested for *AIRE* gene mutations. In our study, 10 APS-1 patients had no detectable mutations in the *AIRE* gene in agreement with other populations [15, 84] and this suggests that not yet identified genes could be involved in the development of APS-1. The *AIRE* gene is strongly implicated in the regulation of organ-specific antigen expression in medullary thymic epithelial cells and in the T cell tolerance [4, 19]. However, as suggested by [86], although *AIRE* is an important transcriptional controller, other controllers should work independently in promiscuous gene expression (PGE). In the light of the foregoing, on a speculative basis, it may be suggested that defects of *AIRE* partners or of other PGE controllers could be responsible for the development of clinical APECED manifestations in patients that do not harbor *AIRE* gene mutations. In future studies, on exome sequencing may be useful in extending our understanding of the genetic causes of APS-1. In previous studies, a correlation between genotype and phenotype has been reported in some patients with APS-1 [6, 20]. For example, in the Norwegian population some gene deletions were associated with the onset of APS-1 later in life and a milder phenotype while some genotypes which lead to formation of truncated proteins appeared to be associated with CMC and AD [20]. In contrast, some patients carrying the same mutation (even siblings) presented different clinical manifestations and experienced different courses of the disease [18, 87]. Among our Italian patients, there were some direct genotype–phenotype associations for R139X, R257X, W78R, T16M and R203X. However, some siblings in our study harboring the same *AIRE* mutation presented remarkably different phenotypes. This suggests that in addition to key *AIRE* gene mutation other genetic, environmental or stochastic factors may impact on the phenotypic expression of APS-1 [20].

Overall, our study is a detailed analysis of all available information on the largest cohort to date of patients with APS-1 from a single national population. As such, this study should enhance the current understanding of the complexity of APS-1 and help in the diagnosis and management of this rare disease ultimately benefiting the affected patients and their families.

## Supplementary Information

Below is the link to the electronic supplementary material.

Supplementary file1 (DOCX 134 KB)

## Abbreviations

17α OHAbs: 17α-Hydroxylase autoantibodies
21-OHAbs: 21-Hydroxylase autoantibodies
AD: Addison’s disease
ACA: Adrenal cortex autoantibodies
AITD: Autoimmune thyroid diseases
AG: Autoimmune gastritis
AIH: Autoimmune hepatitis
*AIRE*: Autoimmune regulator
AIREp: Autoimmune regulator protein
ACD: Autoimmune connective diseases
AADCAbs: Aromatic-l-aminoacid decarboxylase autoantibodies
AIH: Autoimmune hepatitis
AID: Autoimmune intestinal dysfunction
APECED: Autoimmune poly-endocrine-candidiasis-ectodermal-dystrophy
APS-1: Autoimmune polyendocrine syndrome type 1
CD: Celiac disease
CMC: Chronic mucocutaneous candidiasis
CH: Chronic hypoparathyroidism
CT: Chronic thyroiditis
DM-1: Diabetes mellitus type 1
ECE-2Abs: Endothelin-converting enzyme-2 autoantibodies
GADAbs: Glutamic acid decarboxylase autoantibodies
GD: Graves’ disease
GH: Growth hormone
HDAbs: Histidine decarboxylase autoantibodies
HH: Hypergonadotropic hypogonadism
IFNωAbs: Interferon-ω autoantibodies
IFA: Intrinsic factor autoantibodies
ICA: Islet cell autoantibodies
LGLL: Large granular lymphocyte leukemia
LKMAbs: Liver–kidney microsomal autoantibodies
MPCAbs: Melanin-producing cell autoantibodies
MAS-1: Multiple autoimmune syndrome type 1
NALP-5Abs: NACHT leucine-rich protein-5 autoantibodies
PCA: Parietal cell autoantibodies
PA: Pernicious anemia
KCNRGAbs: Potassium channel-regulating autoantibodies
POF: Premature ovarian failure
PGE: Promiscuous gene expression
IA_2_Abs: Second islet antigen autoantibodies
SCCAbs: Side-chain cleavage autoantibodies
SMA: Smooth muscle autoantibodies
SA: Spleen atrophy
SEA: Steroid enzyme autoantibodies
StCA: Steroid-producing cell autoantibodies
TgAbs: Thyroglobulin autoantibodies
TMAbs: Thyroid microsomal autoantibodies
TPOAbs: Thyro-peroxidase autoantibodies
tTgAbs-IgA: Tissue transglutaminase autoantibodies of IgA class
Tr-4Abs: Transglutaminase-4 autoantibodies
TPHAbs: Tryptophan hydroxylase autoantibodies
TSHRAbs: TSH-receptor autoantibodies
