# Supplementary material for: Autoimmune polyendocrine syndrome type 1: an Italian survey on 158 patients
Source: J Endocrinol Invest. 2021 May 18;44(11):2493–510. doi: 10.1007/s40618-021-01585-6 (PMC8502131; doi:10.1007/s40618-021-01585-6)

**Supplementary Table 1** *AIRE* gene mutations in APS-1 patients in different macro-areas or regions of Italy.

| **Regions or Macro-areas** | **Number of**  **patients**  **158** | **Tested**  **for AIRE**  **136** | ***AIRE* gene mutations** | **Most prevalent mutations** |
| --- | --- | --- | --- | --- |
| **North-East**  -Veneto,  -Trentino Alto-Adige  -Friuli-Venezia Giulia | **28** | **25** | 9 cases R257X/R257X  5 cases R257X/C322fsX372  4 cases C322fsX372/C322fsX372  1 case R257X/F318Sfs  1 case 905_906delGT/C322fsX372  1 case R139X/R139X  1 case D312N/negative  1 case P236L/negative  2 cases negative/negative | R257X 48%  C322fsX372 25% |
| **North-West**  -Lombardia  -Piemonte  -Liguria | **14** | **12** | 2 cases C889T/C889T  2 cases L87P/C322fsX372  1 case W78R/361delC  1 case R257X/C322fsX372  1 case 1085-1097del/negative  1 case 1039delC/1085-1097del  1 case R471C/R471C  1 case L87P/P539L  1 case R471C/negative  1 case 239dupT/IVS8+3G>t:+5G>T | C889T 16%  L87P 13%  C322fsX372 13%  R471C 13% |
| **Central**  -Emilia-Romagna  -Marche  -Toscana  -Lazio | **13** | **11** | 2 cases W78R/W78R  1 case T16M/T16M  2 cases c834C>G/negative  1 case G228W/negative **(dominant)**  5 cases negative/negative | W78R 25%  T16M 25%  Negative 59% |
| **South**  -Calabria  -Basilicata | **8** | **7** | 3 cases W78R/W78R  1 case 1085-1097del/1085-1097del  1 case R203X/R203X  1 case A21V/A21V  1 case C322fsX372/C322fsX372 | W78R 43%  R203X 14%  1085-1097del 14% |
| **CAMPANIA** | **15** | **12** | 2 cases W78R/T16M  2 cases IVS1+5delG/IVS1+5delG  2 cases T16M/T16M  1 case W78R/A21V  1 case 62C>T/976-679  1 case c1314-1326del13insGT/c1314-1326del13insGT  1 case T16M/S196S  1 case R471C/negative  1 case S196S/A399A | T16M 29%  IVS1+del5G 17%  W78R 13% |
| **APULIA** | **15** | **15** | 5 cases W78R/W78R  3 cases W78R/Q358X  2 cases T16M/T16M+P252L  1 case W78R/P252L  1 case W78R/D22_V23del  1 case P539L/P539L  1 case C311fsX377/C311fsX377  1 case C311fsX376/p539L | W78R 54%  Q358X 11%  P252L 11% |
| **SICILY** | **20** | **16** | 3 cases R203X/R257X  2 cases R203X/R203X  2 cases R257X/A21V  2 cases IVS13+2insT/IVS13+2insT  1 case R203X/IVS9+5G>t  1 case R257X/W78R  1 case T16M/S107C,Q108sf (new mutations)  1 case A21V/C322fsX372  1 case A21V/W78R  2 case negative/negative | R203X 25%  R257X 19%  A21V 13% |
| **SARDINIA** | **28** | **28** | 24 cases R139X/R139X  2 cases R139X/C322fsX372  1 case R139X/negative  1 case R139X/D304E | R139X 93% |
| **MIXED ORIGIN**  -Sardinia/Lazio (2)  -Sardinia/Marche  -Sardinia/Calabria  -Sicily/Sardinia  -Sicily/Lazio  -Veneto/Lombardy  -Campania/Emilia-Romagna | **8** | **8** | 2 cases R139X/C322fsX372  1 case R257X/C322fsX372  1 case R203X/R139X  1 case T16M/G298L  1 case T16M/A21V  1 case C322fsX372/T16M  1 case negative/negative | C322fsX372 25%  T16M 19%  R139X 19% |
| **UNDEFINED ORIGIN** | **9** | **2** | 1 case R139X/c302fs  1 case negative/negative |  |

**Supplementary Figure 1** The range of reported prevalence of major and minor clinical diseases in 568 APS-1 patients from 20 national cohorts reported between 1998 and 2018. Every bar represents the range from the lowest to the highest reported prevalence of the respective clinical manifestation (for example, Addison’s disease was reported in 22 to 100% of the cohorts).


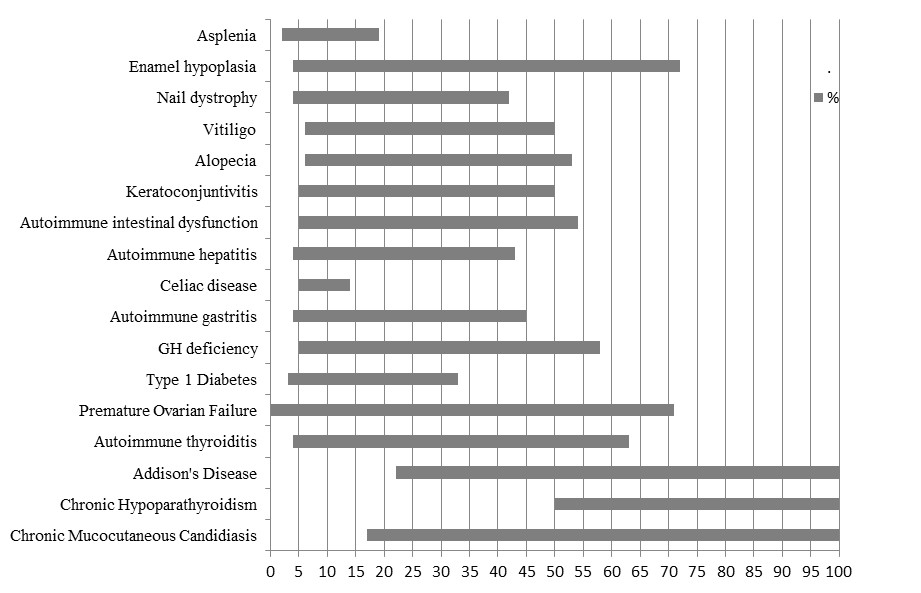

Supplement: Supplementary file 1 — Supplementary file1 (DOCX 134 KB) [file 40618_2021_1585_MOESM1_ESM.docx]
